# Pennsylvania Hospital

**Published:** 1839-09-07

**Authors:** Henry Wheaton Rivers

**Affiliations:** Resident Surgeon


### CLINICAL REPORTS.

### PENNSYLVANIA HOSPITAL.

[Reported by Henry Wheaton Rivers, M.D., Resident Surgeon.]

*List of Accidents admitted into the Pennsylvania Hospital, from August 16th to August 31st, 1839, inclusive.*

A case of simple fracture of the anatomical neck of the humerus, from a fall directly on the front of the shoulder. On first coming into the house, it was found difficult, owing to the swelling which had taken place, to diagnosticate the injury. Leeches were applied to the shoulder, and afterwards soap liniment. By these means, and keeping the patient at perfect rest, the swelling was diminished in the course of three or four days, when the above fracture was detected. The treatment of the fracture consisted in placing the arm in three splints, having first applied a roller from the ends of the fingers up, a pad in the axilla, and the whole confined by a broad bandage around the body. Doing well.

A case of contusion of the right side and left hip, caused by falling from the fourth story of a store, through a hatchway. Treated by six wet cups to side, followed by soap liniment, and, the following day, six wet cups to hip. Doing well.

A case of oblique fracture of the radius, near its lower end, from a fall on the palm of the hand. Treated in the usual way.

A case of gunshot wound, from a pistol discharged by a suicide. The ball entered the left breast, just below and to the left of the nipple of that side, and was cut from the axilla on the following morning, the counter opening being seven inches from the place of entrance. The patient, at the time of his entrance, was labouring under an attack of mania a potu, for which he was treated, and a poultice applied to the wound. Died of erysipelas on the seventh day after the accident.

A case of strangulated inguinal hernia of the right side; reduced in about half an hour. An injection having been given, and the patient put in a warm bath, a truss was accurately applied, and the patient discharged three days after.

A case of laceration of the hand, caused by a piece of coal falling on it. Treated with poultice; afterwards with adhesive strips, and simple cerate.

A case of compound and comminuted fracture of the fore arm; a simple fracture of the humerus of the same side, which rendered amputation necessary; a laceration of the internal lateral ligament of the knee joint, and partial dislocation of the patella; also, a compound fracture of one or more of the tarsal bones of the opposite foot.

A case of concussion of the brain, caused by a fall from the foretopsail yard of a ship, the patient striking on the back of his head. Cold was applied to the head, and mustard plasters to feet; afterwards leeches to back of head. Doing well.

A case of contusion of both feet, caused by the passage of a loaded cart over them. Treated by leeches, cold applications, and keeping the patient at rest, with the feet elevated.

A case of scalded foot; treated by lime water and olive oil. Doing well.

A case of contusion of the back, from falling down stairs; treated by rest, and six wet cups to part.

A case of compound and comminuted fracture of the left foot, caused by the passage of a railroad car over it. This case was admitted last night, and amputation was performed by Dr. Norris, this morning, (Sept. 1st,) below the knee. The patient has reacted.

---

*Case of Gangrene of the Penis, from Syphilis—Discharged cured in nineteen days.*

A. H., aged twenty-nine years, admitted August 5th, 1839. About two weeks previous to entering the house he first perceived a small chancre on prepuce, behind the glans penis. Did nothing for it until five days after, during which time it increased in size rapidly. On the fifth day he took a dose of calomel and rhubarb, which operated on his bowels freely; continued taking the calomel and rhubarb daily for some time, each dose operating on bowels; was advised to follow this course by a student of medicine. He then applied to a quack, under whose treatment he remained for several days. The penis, at the time of his entrance, was in a state of gangrene for about two inches. Ordered fomenting poultice to be applied to whole penis, syr. sarsap. comp. f℥ss. thrice daily; pulse one hundred; bowels open; passes urine freely.

*August 6th.*—Slept badly last night. Ordered liq. morph. sulph. f℥iss. thrice daily. Diet—soup, vegetables, tea, bread.

*7th.*—Slept well; penis still sloughing; some sloughs came away; bowels open; pulse ninety-six; continue poultice; slight hæmorrhage this afternoon, easily suppressed with a dossil of lint. Continue treatment as before.

*8th.*—Did not sleep very well last night; pulse ninety-two; tongue furred; bowels open; sloughing continues, and upon passing a probe into the urethra, it was found to come out from the back part of the penis a few lines below the glans. Ordered a half bottle of porter daily.

℞. Quin. Sulph. gr. xii.
Acid. Sulph. gtt. v.
Aquæ Cinnam. f℥ijss.
M. S. f℥i. q. t. h.

Flaxseed poultice to penis at bedtime.

*9th.*—Slept very well; sloughs coming away; passes most of urine from the opening in back of penis; pulse eighty; tongue slightly coated; bowels open; low spirited. Diet—soup, rice, fruit.

*10th.*—Slept well in the latter part of the night; pulse seventy-two, soft; tongue furred; bowels open; gangrenous part nearly separated; taken away with scissors.

*11th.*—Rested badly; tongue cleaner; bowels open; pulse seventy-six; perspires a great deal in sleep, and awakes in a chill; stump of penis looks well; touched with a wash composed of acid. nit. gtt. xv., aquæ f℥iv., daily; flaxseed poultice continued; other treatment as before.

*12th.*—Rested badly; pulse seventy-six; tongue cleaner; penis improving. Ordered

℞. Kreosote gtt. vi.

Cret. ppt. ʒij.

Axung. ℥i.

M. S., apply to penis. Diet as before.

*13th.*—Rested well. Tongue clean; bowels open; pulse sixty-eight; says he feels almost well; edges of stump touched with argent. nit.; cicatrizing rapidly; no contraction of urethra.

*20th.*—Stump cicatrized.

*24th.*—Discharged cured.

---

*Case of Compound Fracture of the Skull, with loss of a portion of Brain—Death sixty days after the accident.*

John Flood, aged sixteen years, admitted July 1st, 1839, at 11 o'clock, A. M. About two hours previous to his entrance he had his head jammed between two portions of a machine used for planing or grooving iron, driven by steam power. A screw about six inches long, to which was attached a head of seven-eighths of an inch in diameter, was driven through the frontal bone, about an inch above the left orbicular ridge, with so much force, and to such a depth, (an inch and a half,) that his fellow workmen were unable to extract it, owing to the head of the screw being beneath the inner table, and the edges of the fracture retaining it there. Surgical aid was called in, and by means of an incision through the integuments, and considerable force, it was at length extracted. The moment of his entrance he was seized with a violent fit of vomiting, mostly of a watery fluid. The brain was found to be protruding; he had very little hæmorrhage. He was immediately put upon a fracture-bed, and cold cloths ordered to be applied to head. About 1 o'clock he had a convulsion, which lasted but a few minutes; after which he became quiet, retaining his intellect perfectly. Not having passed his urine at 9 P. M., and complaining of pain in the region of the bladder, hot tins were applied over the abdomen, soon after which he succeeded in voiding a quantity of urine of a natural appearance.

*July 2d.*—Slept pretty well. Has paralysis of right *arm*. Diet, barley water and arrowroot. A small portion of brain came away with dressings. Ordered cold to be continued to head, and V. S. f℥viij., but was able to obtain but f℥iij.

*3d.*—Passed a good night; says he feels pretty well, with the exception of his arm; pulse eighty; tongue slightly furred. Having had no evacuation from bowels, ordered a dose of salts and magnesia, which was immediately ejected. Ordered

℞. Hyd. Prot. Chlor.

Ex. Col. Comp. āā gr. v.

A common injection was also ordered, which was followed by a copious evacuation and much relief.

*Night.*—Some fever; pulse ninety; tongue slightly furred; slight erysipelas about wound. Ordered V. S. f℥viij., and

℞. Mist. Neut. f℥vi.

Ant. Tart. gr. i.

M. S., f℥ss., q. t. h.

This mixture producing nausea, the ant. tart. was omitted, and the mist. neut. continued.

*4th.*—Slept well last night. Continued comfortable through the day. An ineffectual attempt was made to take blood in the evening.

*5th.*—Rested well last night. Ordered

℞. Hyd. Prot. Chlor.

Ex. Col. Comp. āā gr. v.

Ft. Pil. No. ij.

S. Decub.

To be repeated every other night.

*6th.*—Rested well; pills operated; skin cool and moist; pulse sixty; tongue clean; continue cold to head, and neutral mixture.

*7th.*—Rested well; some sloughs came away; wound looks well.

*12th.*—Slept well; bowels open; towards night complains of pain shooting across the forehead; pulse eighty; skin cool. Continue treatment as before.

*13th.*—Passed a bad night; pain across both eyes.

*14th.*—Slept well; pain continues over eyes; wound discharging; bowels open; skin cool; tongue clean; pulse sixty; respiration more laboured.

*17th.*—Wound looking well; discharge continued. Ordered

℞. Pil. Hyd. gr. iij., thrice daily.

*19th.*—Slept well; motion returning in right arm; pulse sixty; tongue natural; skin cool and moist; wound cicatrizing; edges touched with argent. nit.

*22d.*—Passed a good night. Wound continues to discharge; lower part of wound drawn together with adhesive strips; regained power over right arm.

*26th.*—Treatment continued the same since last date; discharge from wound continues; edges touched with argent. nit. daily.

*28th.*—Complains of pain in head; sleeps well at night; pupils natural; evening pain gone. Diet, barley water, arrowroot, gruel, with a cracker broken it.

*August 4th.*—Doing well; compress to lower part of wound, with adhesive strips; argent nit. to edges; continue cold to head, and pil. hyd.; mouth not touched.

*6th.*—Rested well; wound diminished in size. Ordered lint wet with aq. cal. applied to wound. Bowels open; pulse sixty; skin cool.

*Evening.*—Pulse ninety-eight; tongue clean; continue neutral mixture; discontinue blue pill during the day,—take one at bedtime; gums spongy.

*10th.*—Sleeps well at night; bowels open; tongue clean; pulse eighty; discharge increased considerably. Ordered a small quantity of soup at dinner time.

*18th—Night.* Complains of pain in head; face flushed; pulse one hundred and twenty. Ordered cold to head to be continued, and neutral mixture.

*19th.*—Had a chill last night, and another this morning; diarrhœa; pulse one hundred and sixteen, frequent. Ordered V. S. ℥viij. Diet, barley water. Poultice to head.

*21st.*—No bad symptom since last date; pulse sixty-eight; tongue slightly coated. Diet, barley water, gruel, and crackers.

*25th.*—Face flushed; pulse ninety-two; tongue coated; bowels open; complains of pain in head.

*26th.*—Pulse ninety-two; skin cool and moist; bowels open; pain in head.

*Night.*—Pulse one hundred and four; skin covered with a cool perspiration; complains of pain all over; no discharge from wound; tongue furred.

*27th.*—Pain in head; tongue furred; slight discharge from wound; pulse sixty; bowels open; partial loss of memory.

*28th.*—Sleeps a great deal; wound the same; bowels not open; tongue furred; pain in head; pulse fifty, irregular. Ordered a dose of salts and magnesia; poultice continued to wound.

*Night.*—Pulse ninety-two, frequent.

*29th.*—Complains of pain in head, some stupor, and delirium; no evacuation from bowels. Ordered an injection, which operated. 12 o'clock, M., comatose; stertorous breathing; pulse 54; extremities cold; left pupil very much dilated; the right contracted. Continued in this state until one o'clock on the morning of the 30th, when he died, sixty days after the accident.

Autopsy nine hours after death. The wound in the skull was found to be about an inch in diameter; a small piece of bone was found driven in and pressing on the brain; dura mater thickened, and closely adherent to brain. The anterior lobe of the left hemisphere of the brain was found to be discoloured, bluish yellow, much softened, and presented evident fluctuation beneath; vessels of pia mater generally distended. An abscess, having no external opening, was found immediately beneath dura mater, occupying the anterior lobe, and filled with about f℥vi. of thick, yellow pus; walls of abscess of an ashy-grey colour, lined with a false membrane of a hard consistence, and about a line and a half in thickness; brain around of a yellowish colour, and much softened. A piece of bone, about an inch and a quarter in length, was found driven beneath the sound bone at the upper part of the wound; at the side of it a small portion was found, strongly adherent to the internal table. The opposite hemisphere of brain was natural; ventricles much distended with serum. The edges of the fractured bone were found to have become rounded off. No further examination could be obtained.

